# Platelet Lysate-Derived Neuropeptide y Influences Migration and Angiogenesis of Human Adipose Tissue-Derived Stromal Cells

**DOI:** 10.1038/s41598-018-32623-8

**Published:** 2018-09-25

**Authors:** Rita Businaro, Eleonora Scaccia, Antonella Bordin, Francesca Pagano, Mariangela Corsi, Camilla Siciliano, Raffaele Capoano, Eugenio Procaccini, Bruno Salvati, Vincenzo Petrozza, Pierangela Totta, Maria Teresa Vietri, Giacomo Frati, Elena De Falco

**Affiliations:** 1 Department of Medico-surgical Sciences and Biotechnologies, Sapienza University of Rome, C.so della Repubblica 79, 04100 Latina, Italy; 2 Department of Surgical Sciences, Sapienza University of Rome, V.le del Policlinico 155, 00161 Rome, Italy; 3 Breast Unit, A.O. U. Università della Campania Luigi Vanvitelli, piazza Luigi Miraglia, 280138 Naples, Italy; 4 Futura Stem Cells Via G. Peroni 400, Rome, Italy; 5 Department of Biochemistry, Biophysics and General Pathology, Second University of Naples, Via Luigi De Crecchio 7, 80138 Naples, Italy; 6 Department of AngioCardioNeurology, IRCCS NeuroMed, 86077 Pozzilli, (IS) Italy

## Abstract

Neuropeptide Y (NPY), a powerful neurotransmitter of the central nervous system, is a key regulator of angiogenesis and biology of adipose depots. Intriguingly, its peripheral vascular and angiogenic powerful activity is strictly associated to platelets, which are source of clinical hemoderivates, such as platelet lysate (PL), routinely employed in several clinical applications as wound healing, and to preserve *ex vivo* the progenitor properties of the adipose stromal cells pool. So far, the presence of NPY in PL and its biological effects on the adipose stromal cell fraction (ASCs) have never been investigated. Here, we aimed to identify endogenous sources of NPY such as PL-based preparations and to investigate which biological properties PL-derived NPY is able to exert on ASCs. The results show that PL contains a high amount of NPY, which is in part also excreted by ASCs when stimulated with PL. The protein levels of the three main NPY subtype receptors (Y1, Y2, Y5) are unaltered by stimulation of ASCs with PL, but their inhibition through selective pharmacological antagonists, considerably enhances migration, and a parallel reduction of angiogenic features of ASCs including decrease in VEGF mRNA and intracellular calcium levels, both downstream targets of NPY. The expression of VEGF and NPY is enhanced within the sites of neovascularisation of difficult wounds in patients after treatment with leuco-platelet concentrates. Our data highlight the presence of NPY in PL preparations and its peripheral effects on adipose progenitors.

## Introduction

Neuropeptide Y (NPY) is a 36-amino acid peptide produced by the central nervous system and implicated in physiological neuron-mediated activities such as food intake or circadian rhythm^1,2^. In the autonomous nervous system NPY is stored in macrovesicles close to the varicosities present along the end terminals of sympathetic nerve fibres, where it is located in association with the adrenergic mediators. NPY is the most abundant neuropeptide in the heart and brain and it is released during nerve activity as well as during ischemia, leading to vasoconstriction and smooth muscle cell proliferation^3^. Importantly, NPY receptors are heterogeneously expressed depending on the tissue of origin and cell type^4,5^. Specifically, Y1, Y2 and Y5 receptors are able to exert differentiated roles in stromal/mesenchymal progenitors compared to endothelial or neural cells, where they are reported to prevent neural death upon cytotoxic insults^5,6^. The Y5 receptor is mainly associated with proliferative and anti-aging properties in bone marrow-derived mesenchymal stem cells (BMMSCs)^7^, whereas Y1 and Y2 are known to influence the differentiation of stromal cells into the osteoblast-adipocyte lineage in vertebrates^8–10^, as well as in capillary angiogenesis^11^. Despite this heterogeneity, Y1, Y2 and Y5 receptors knockout mice similarly exhibit higher body weight, increased white adipose tissue and alteration of the cardiometabolic traits, suggesting that a multi- and combined receptor action exists^12^. Additionally, NPY exerts an anti-fibrotic potential in the ischemic heart^1,13^, thus contributing to tissue remodelling in the injured cardiac tissue. More importantly, NPY mediates the angiogenic process by enhancing endothelial cell proliferation in a calcium-dependent manner^14^. Intriguingly, it has been recently reported that platelets produce NPY, which is critical to mediate neo-angiogenesis upon *in vivo* ischemic insult^15^, exhibiting vascular activity comparable to that exerted by acknowledged angiogenic soluble mediators such as basic FGF or VEGF^16–18^. To date, regenerative medicine is focused on the identification of factors involved in the recruitment of stem cells to the site of injury and on molecules promoting maturation processes of undifferentiated progenitors.

In view of that, platelet lysate (PL), defined as an hemoderivate of platelets origin, is enriched with plethora of growth factors and biologically active mitogens^19,20^ and it is currently and safely employed in several clinical applications, in *in vivo* studies and as *in vitro* substitute to foetal bovine serum for the *ex vivo* expansion of the adipose stromal fraction^21^. Platelet lysate is known to enhance angiogenesis, a key feature for tissue injuries such as dermal wounds and ulcers, where the employment of PL or autologous leuco-platelet concentrate has been successfully consolidated^14,15,22^. Importantly, the restorative activity of PL both *in vivo* and *in vitro* consists in the enhancement of defined biological properties such as proliferation, migration and preservation of the clonogenic ability combined to changes of the cellular and phenotypic plasticity^14,20–23^. PL is also described to influence the biological behaviour of multiple adult cell populations^19,20,22,24^. The adipose stromal fraction represents the most reactive tissue source to PL. A large number of reports have clearly demonstrated the biological advantages and the efficacy of PL on the adipose tissue-derived stromal cells (ASCs), including modifications of the ASC-derived secretome, angiogenesis and differentiation capacity^14,20–22,25^.

Similarly to PL, NPY is either involved in the promotion of adipogenesis and inflammation and in the peripheral vasoconstriction/angiogenesis^8,11,23,26^, including wound healing^10,27^. More importantly, the mesenchymal fraction is a target of NPY, as demonstrated in the bone marrow niche, whose homeostasis is strictly controlled by NPY^28^.

The presence of NPY in PL-based preparations can be only indirectly assumed, as it has never been proven. Consequently, the biological and molecular mechanisms mediating PL-derived NPY action are still to be fully unravelled. Notably, Tilan *et al*. have demonstrated that during the late stages of ischemia in a murine model, systemic levels of NPY are enhanced in platelets by circulating megakaryocytes, therefore boosting capillary angiogenesis and vessel maturation at the sites of neovascularization^11^. This study has significantly highlighted the close dependence between NPY originated from platelets and peripheral vascular/angiogenic effects.

Accordingly, we investigated if PL may be identified as endogenous source of NPY with bio-activating properties, and in particular if NPY may contribute as endogenous mediator to the intrinsic biological properties of PL on the human adipose stromal fraction, routinely expanded *ex vivo* using this hemoderivate.

Our findings highlight that PL contains a high amount of endogenous NPY, which is able to significantly modulate ASCs angiogenesis and chemotaxis. Moreover, the blocking of NPY action through the selective Y1, Y2 and Y5 antagonist receptors inhibition, significantly downregulates intracellular calcium levels in ASCs stimulated with PL, without impacting on ERK signalling pathway and nitric oxide (NO) levels.

Overall, our data demonstrate the contribution of PL-derived NPY in mediating both angiogenesis and migration of human ASCs, identifying a potential calcium-dependent mechanism of action and elucidating the somatotropic and biological effects of NPY.

## Materials and Methods

### Isolation, expansion and *in vitro* stimulation of adipose stromal cells (ASCs)

Human adipose stromal cells (ASCs) were isolated from subcutaneous depots as we previously reported^20–22^. Briefly, biopsies were washed with PBS without Ca^2+^/Mg^2+^ and erythrocytes were lysed (Qiagen, Hilden, Germany). Samples were then mechanically chopped and enzymatically digested in Trypsin/EDTA and 1 mg/ml Collagenase I (Gibco, Invitrogen) for 45 minutes at 37 °C. Samples were then filtered and serially spun at 1200 rpm for 5 minutes. ASC cultures were seeded at 4000 cells/cm^2^ in DMEM-Low glucose in 10% Fetal Bovine Serum (FBS)/200mM L-glutammine/100 mM sodium pyruvate/non-essential aminoacids/penicillin/Amphotericin. After 3 days, the whole media was removed and the cultures expanded until reaching subconfluence. Viability was performed by Trypan Blue exclusion assay. At passage 3, cells were starved with 0.2%PL (patented GMP-compliant Platelet Lysate, Mesengen, Italy)^14,19–22^ overnight, then stimulated with DMEM-Low glucose/10%PL ± NPY subtype receptors antagonists (0.01 M Y1, BIBP3226 Cat. N. 2707; 0.025 M Y2, BIIE0246 Cat. N. 1700; 0.1 M Y5, L-152,804 Cat. N. 1382, all Tocris) for 48 hours. Procedures performed in this study involving human participants were in accordance with the ethical standards of the institutional Ethical Committee Policlinico Umberto I (Prot. 688/11 Rif. 2229/21.07.2011) and with the 1964 Helsinki declaration and its later amendments or comparable ethical standards. Informed consent was obtained from all individual participants included in the study.

### Clonogenic and adipogenic differentiation assays

Clonogenic potential has been performed as already described^20,22^. Briefly, secondary colonies were obtained by seeding at low density (10 cells/cm^2^) in complete medium with 10%PL and ± NPY subtype receptor antagonists. Cultures were incubated for 14 days at 37 °C. Colonies produced were fixed with 4% paraformaldehyde and then stained with Giemsa (Sigma, Milan, Italy) for 1 hour and counted by optical microscope. A cluster of >50 cells was considered as a colony. The Colony forming efficiency (%) was calculated as ratio: N. secondary colonies/N. plated cells x 100. For adipogenic differentiation tests ASCs (1 × 10^4^ cells/cm^2^ were cultured for 3 weeks using StemPro® Adipogenesis Differentiation kit (GIBCO, Grand Island, NY, USA, Cat. N. A10070-01)^20,22^. Accumulation of lipid droplets was evaluated by Oil Red Oil staining (Sigma-Aldrich, St. Louis, MO, USA, Cat. N. 01391-500 ml). Adipogenesis was quantified in 10 randomly selected fields observed under an optical microscope. The number of positive adipocytes was counted. The percentage of adipogenic differentiation was calculated as following: (N. adipogenic cells/N. Total cells) x 100.

### Migration assay

After starvation human ASCs were resuspended in DMEM serum free ± Y1, Y2 and Y5 receptor antagonists and seeded at 5 × 10^4^/well in the upper side of 96 well Boyden chamber (8 µm pores, BD Biosciences). The lower chamber contained DMEM or 10% PL ± Y1, Y2 and Y5 receptor antagonists. Cells were incubated at 37 °C for 16 hours. Afterwards, the inner side of the insert was wiped with a wet swab, in order to remove the cells and the outer side of the insert gently rinsed with PBS, fixed with methanol for 20 minutes and stained with 0.25% Giemsa (Sigma, St. Louis, USA) for 30 minutes, rinsed again, and dried. Migrated cells were counted (ten fields per chamber) under an optical microscope. Experiments were repeated three times in duplicate.

### Proliferation assay

Cell proliferation was evaluated by MTS assay as already described^19^ by employing the CellTiter 96 AQueus One Solution Cell Proliferation Assay (Promega, Milan, Italy) and according to manufacturer’s instructions. After starvation (0.2% PL), ASCs were seeded in 96 well plates (cell density of 6 × 10^3^ cells/well) in quintuplicate and conditioned for 48 hours with 10% PL ± NPY subtype receptor antagonists. The stimulation with recombinant NPY (10^−9^ M, Tocris Cat. N. 1153) was considered as biological control. The optical density signal measured at 492 nm was normalized to that of day 0 after cell seeding.

### *In vitro* angiogenesis

Adipose stromal cells-derived angiogenesis was evaluated by Matrigel assay. Fifty microliters of Matrigel matrix growth factor reduced (BD, San Jose, CA, USA, Cat. N. 356231) were layered in 96-well plates and incubated for 1 h at 37 °C. After treatment with DMEM (unstimulated control) or 10% PL ± NPY subtype receptor antagonists, ASCs were then trypsinized and seeded on the top of the solidified Matrigel (1 × 10^4^ cells/well). The cultures were incubated at 37 °C and after 2 hours, cells were checked under an optical microscope then photographed. Parameters including number of loops and network length were quantified by ImageJ Angiogenesis Analyser Software (National Institutes of Health, USA).

### ELISA assay

Soluble levels of NPY in undiluted PL preparations, 10% PL fractions, DMEM (used as negative control) and ASC-derived conditioned media have been detected by ELISA assay (Elabscience, Cat. No. E-EL-H1893) according to manufacturer’s instruction.

### Western Blotting

Cells were washed and lysed for 30 minutes at +4 °C in equal volumes of ice-cold RIPA lysis buffer (10 mM Tris pH 8, 1%Triton, 0,1% SDS, 0,1% Deoxycholate, 140 mM NaCl, 1 mM EDTA) and a cocktail of protease inhibitors (1 mM DTT, 1 mM PMSF, 1 Protease inhibitor tablet/10 mL Sigma, St. Louis, USA). Homogenates were incubated in ice for 20 minutes, then clarified by centrifugation at 14460 g for 15 minutes. Supernatants were boiled for 5 minutes in Laemmli sample buffer. Protein concentration was determined by Bradford and then separated by 15% SDS-PAGE electrophoresis and electrotransferred to polyvinylidene difluoride membranes. Incubation was performed overnight with the primary antibodies: Rabbit anti-Y1 and Rabbit anti-Y2 (1:500, Bioss Cat. N. bs-1070R and Cat. N. bs-0937R, respectively), Rabbit anti-Y5 (1:500, Abcam Cat. N. ab43824), Rabbit Anti-NPY (1:1000), phospho-ERK1/2 (1:200) and ERK (1:200; all Cell Signaling Technology, Cat. N. 11976 S, 1:1000, Cat. N. #4695, Cat. N. #9106, respectively) and a horseradish peroxidase-conjugated rabbit secondary antibody (Amersham mouse Cat. N. NA93IVS, Amersham rabbit NA934VS). The membranes were developed by enhanced chemiluminescence kit (ECL, GE Healthcare Bio-Science, Piscataway, NJ, USA). Acquisition and densitometry analysis was performed by ChemiDoc and Image Lab 5.2.1 (Biorad).

### Immunofluorescence

In order to detect the expression of Y1, Y2 and Y5 on treated ASCs, cells were fixed with 4% Paraformaldehyde for 15–20 minutes at room temperature then washed 3X with PBS. Cells were then washed again with PBS, then resuspended in blocking buffer (0.25%gelatin/4%FBS) for 1 hour at room temperature. Primary antibodies (Anti-Y1 Bioss bs-1070R; Anti-Y2 Bioss bs-0937R; Anti-Y5 Abcam Cat. N. ab43824. All diluted 1:100) were incubated overnight in 0.25% gelatin at 4 °C. Goat Anti-Rabbit IgG (H + L) (Highly Cross-Adsorbed Secondary antibodies, Alexa Fluor-488, Thermo Fisher, Cat. No. #A-11034) for 45 minutes at room temperature in the dark were added after washing in PBS. Nuclei were stained with DAPI (4′−6′-Diamidino-2-phenylindole, powder ≥98%; Sigma, Milan, Italy Cat. N. D9542). Images were visualized and images acquired by Nikon Eclipse.

### Real Time PCR

Total RNA was isolated (RNeasy kit, Qiagen) and c-DNA obtained and amplified by the SensiMix SYBR Hi-ROX kit (Bioline, London, UK). Templates were amplified by the 7900HT Fast Real Time PCR System (Applied Biosystems, Cheshire, UK) for 40 cycles according to the following protocol: 95 °C for 15 seconds, 60 °C for 10 seconds, 72 °C for 30 seconds. Primer sequences used are: NPY forward GCGACACTACATCAACCTCATC; NPY reverse TGTGCTTTCTCTCATCAAGAGG; VEGF forward AAAAACGAAAGCGCAAGAAA; VEGF reverse TTTCTCCGCTCTGAACAAGG; GAPDH forward ACAGTCAGCCGCATCTTC; GAPDH reverse GCCCAATACGACCAAATCC. GAPDH was considered as housekeeping. The reaction products were analysed by SDS 2.1.1 Software (Applied Biosystems, Cheshire, UK).

### Nitric oxide (NO) levels

NO production in ASC-derived supernatants was evaluated by measurement of nitrite, a stable end product of NO. Nitrite was determined by a colorimetric assay with Griess reagent. One-hundred microliter of supernatant reacted with an equal volume of Griess reagent (one part of 1% sulfanilamide dissolved in 0.5 M HCl and one part of 0.1% N-(1-napthyl)-ethylenediamine dihydrocloride dissolved in distilled water) in 96-well plate under reduced light at room temperature during 10 minutes. The absorbance was measured with a microplate reader at 545 nm using a calibration curve of sodium nitrite standards (0–100 μM).

### Calcium detection

After starvation, ASCs were trypsinized and washed in PBS and incubated with the treatments and the staining solution (3 μM Fluo-4-AM/2.5 mM Probenicide/3% DMSO, all Thermo Fisher) for 20 minutes at 37 °C. After incubation, cells were washed 2X with PBS and then resuspended with cold FACS buffer (PBS/2% FBS). Cells stimulated with Ionomycin (1 μM), EDTA (20 mM) or recombinant NPY (10^−9^ M) were used as positive, negative and biological internal control, respectively. Cytometry was performed using FACSAria II (B&D, San Jose, USA) and data were acquired and analysed by Software DiVa (v6.1.1, B&D, San Jose, USA).

### Immunoistochemistry

Immunohistochemistry analysis was performed on paraffin-embedded consecutive sections (3 μm thickness) from difficult wounds. After deparaffinization with graded concentrations of xylene and alcohol, rehydration and blocking of endogenous peroxidases, samples were firstly incubated in 0.1% Trypsin/PBS and then with serum for 30 and 10 minutes, respectively. Primary antibodies Rabbit Anti-NPY (Cell Signaling Technology, Cat. N. 11976 S 1:100), human VEGF (Abcam, Cat. N. Ab46154, 1:200) or CD31 (Novocastra Leica, Cat N. NCL-CD31-1A10, 1:100) were incubated at 4 °C overnight. The staining was revealed by the avidin-biotin immunoperoxidase complex (Vectastain ABC kit, Vector Laboratories, Peterborough, UK) for single staining. For colocalization studies (NPY/CD31) secondary antibodies were the following: Ultratek HRP (Scytek Laboratories, Cat. N. UHM125 and AFK600) and HighDef Blue IHC chromogen (Enzo, Cat. N. ADI-950-151-0030) and AEC substrate kit (Scytek Laboratories, Cat. N. ACG500). Negative controls consisted of the secondary antibody alone and the use of the Immunoglobulin from the same animal species of the primary antibodies. Mayer’s haematoxylin was used as nuclear counterstain. The Images of the samples were analyzed under a light microscope (Nikon Eclipse). At least 4 random fields were acquired and quantified by identifying the foci of angiogenesis. Results have been expressed as the following ratio = Number of positive foci of angiogenesis/Number total foci of angiogenesis and normalized *vs* time 0.

### Statistical Analysis

Statistical Analysis was performed by using software GraphPad Prism 5 software (San Diego, USA). Comparison between two or more groups was performed by t test or ANOVA (Bonferroni correction or Fisher LSD post-test), respectively. A p value < 0.05 has been considered statistically significant. Data are presented as mean ± standard error unless specified.

## Results

**Figure 1:**
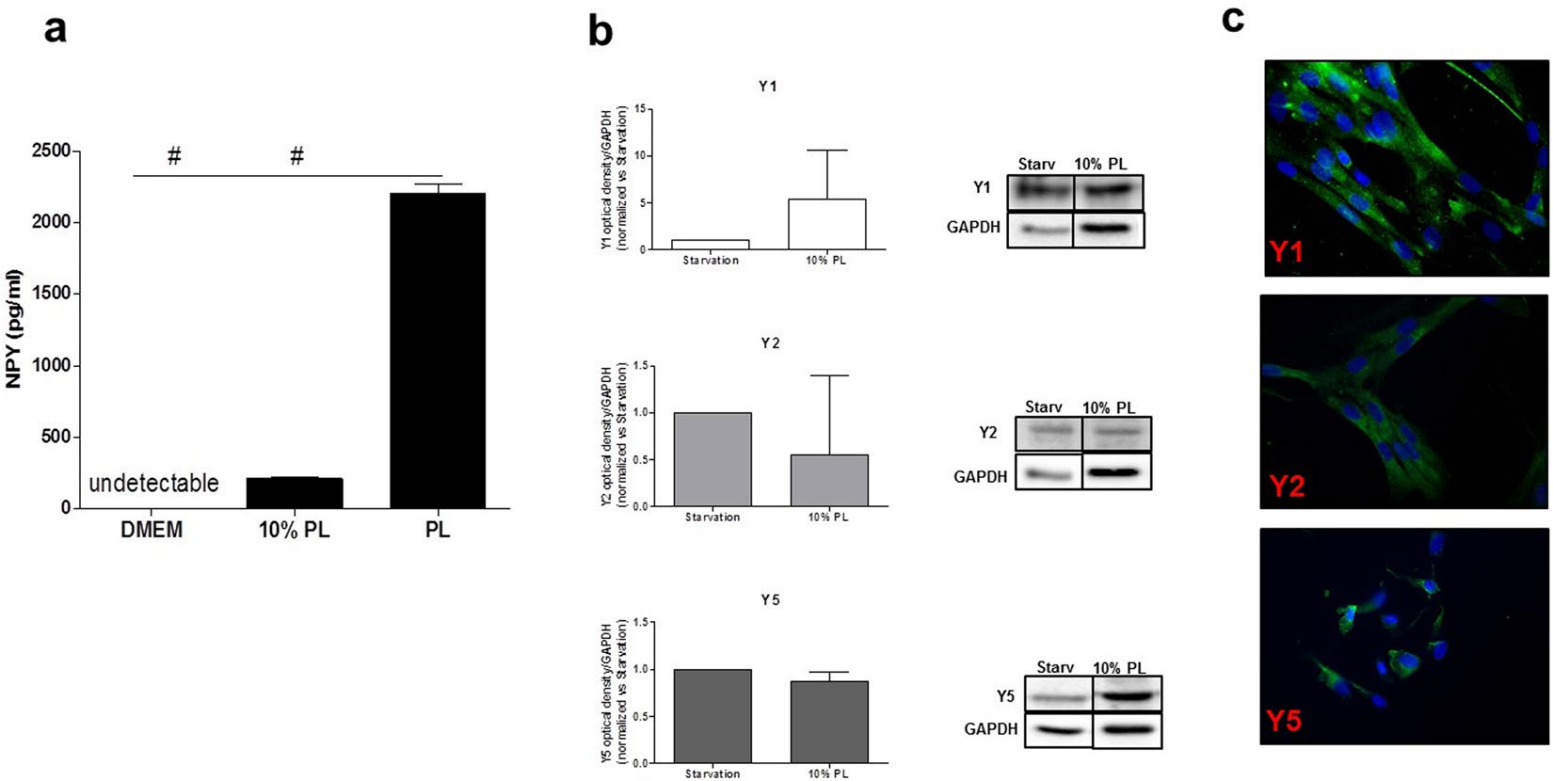
(**a**–**c**) ELISA assay for the detection of soluble human NPY levels in PL-based preparations and expression of Y1, Y2 and Y5 receptors on ASC cultures. (**a**) The graph shows the amount of NPY contained in undiluted platelet lysate preparations as well as in 10% PL. DMEM has been used as negative control. N = 5). PL, platelet lysate. ^#^p < 0.0001. (**b**) Western blot analysis shows that the stimulation with 10% PL for 48 hours does not alter the protein levels of each of the three main receptors for NPY. GAPDH was used as loading control. N = 3. Cropped images are from the same samples split on three different gel runs (Y1, Y2, Y5). Full-length blots are shown in Supplementary Fig. 1d. (**c**) Immunofluorescence for Y1, Y2 and Y5 on ASCs cultures confirms the expression of the three main receptors for NPY. Magnification 20×. PL, platelet lysate. DAPI (blue, nuclei); Y1, Y2, Y5 receptors (green).

We have investigated whether PL preparations contain a source of endogenous NPY, and to this aim we have quantified soluble NPY levels in five different batches of PL by ELISA. Results have shown that undiluted PL-based preparations contain the highest amount of NPY (Fig. 1a, p < 0.0001vs both DMEM and 10% PL), which is still quantifiable in the corresponding 10% PL preparations (the percentage of PL provided as supplement to ASCs cultures in the following experiments). The levels of NPY in the sole DMEM were undetectable (Fig. 1a).

In light of this observation, we have explored the contribution of NPY in PL-mediated effects in ASCs expansion, routinely performed *ex viv*o using PL in the culture media^20–22^. Thus, after isolation of human ASCs, cultures have been stimulated with 10% PL for 48 hours. To explore whether treatment with PL was able to influence the levels of the three main functional active NPY receptors (Y1, Y2 and Y5)^29,30^, we have performed a Western blot analysis. Results have displayed that the stimulation with PL was unable to modulate the protein levels of any of three main NPY receptors (Fig. 1b, Y1 p = 0.45, Y2 p = 0.62, Y5 p = 0.25). The presence of the three main receptors for NPY was confirmed by immunofluorescence. The cellular distribution of NPY receptors on the ASCs cultures showed a distinct and punctuate localization of the Y5 receptor respect to Y1 and Y2, both displaying a more diffuse pattern (Fig. 1c).

**Figure 2:**
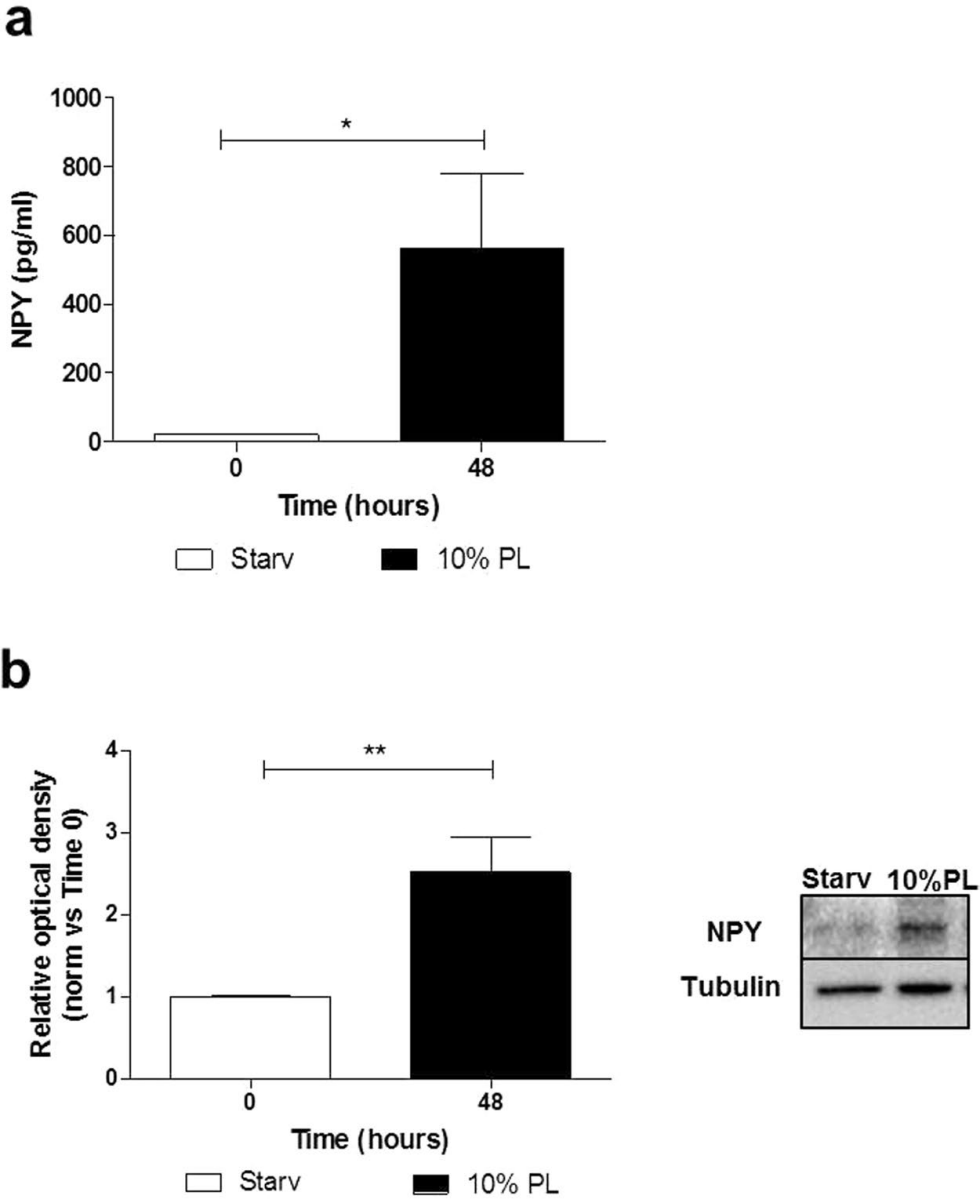
(**a**,**b**) NPY amount from ASCs and ASC-conditioned media (**a**) ELISA assay for soluble human NPY. The levels of excreted NPY in ASC-conditioned media after 48 hours of stimulation with 10% PL are significantly higher compared to starvation. PL, platelet lysate; starv, starvation. *p < 0.05. N = 5. (**b**) Western blot analysis for NPY in ASCs. After 48 hours of stimulation with 10% PL, ASC cultures exhibit enhanced protein levels of NPY. Tubulin is the loading control. Cropped images are from samples ran on the same gel. Full-length blots are shown in Supplementary Fig. 2c. N = 4. PL, platelet lysate; starv, starvation. *p < 0.05.

In order to understand the potential contribution of ASCs in sourcing NPY beyond PL and considering that the 10% PL media provided a considerable amount of soluble NPY (Fig. 1a), we stimulated ASC cultures with 10% PL for 48 hours and evaluated possible alterations of soluble levels of NPY in ASC-derived conditioned media. Results have shown a significant increased extracellular release of the neuropeptide compared to starved cultures (Fig. 2a, p = 0.036). This result was coherent with the enhancement of NPY protein levels in ASC cultures (Fig. 2b, p = 0.012), therefore strengthening the hypothesis regarding the contribution of ASCs in sourcing NPY in the microenvironment beyond the endogenous amount in PL-based preparations.

**Figure 3:**
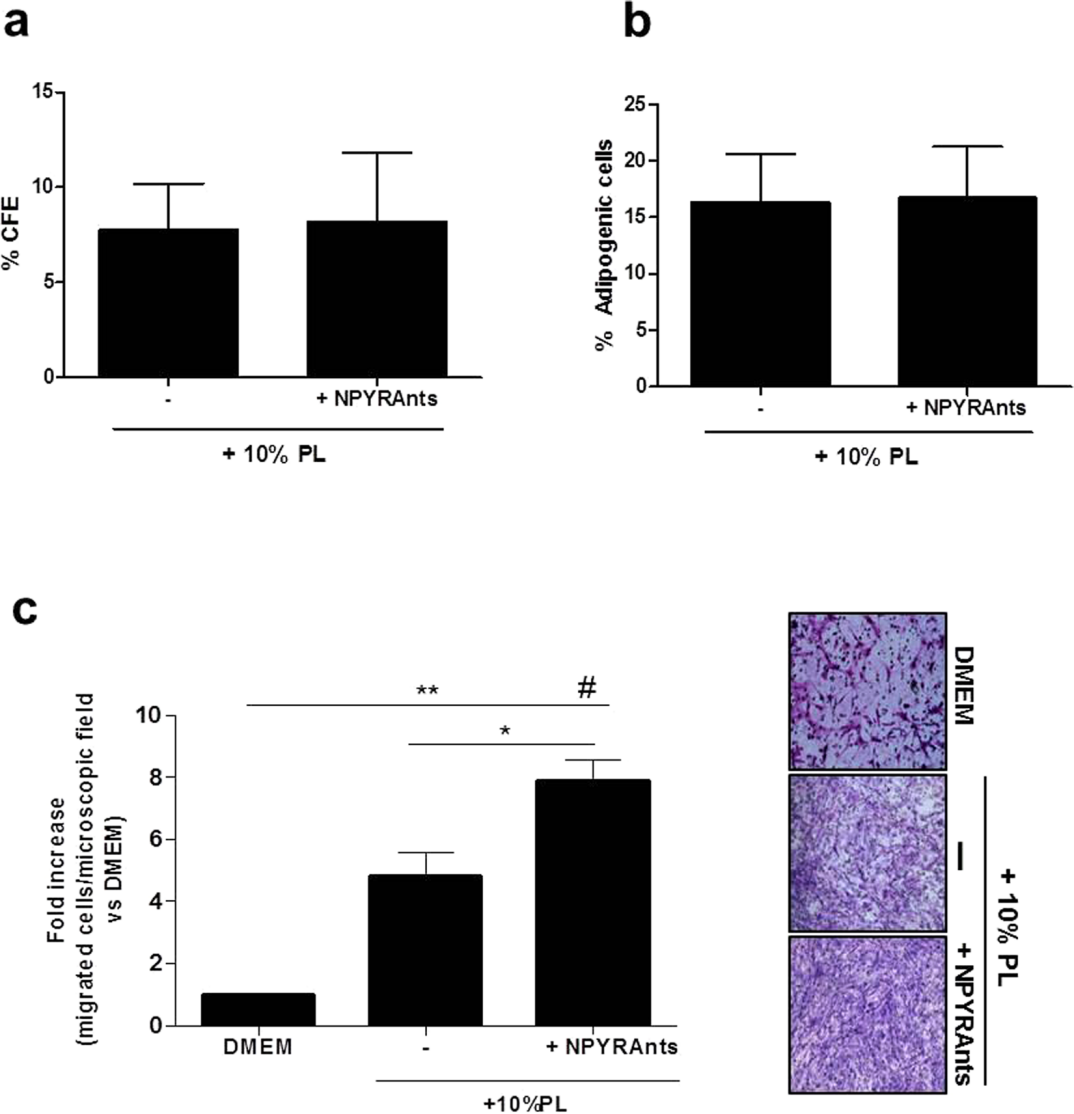
(**a**–**c**) ASCs clonogenic activity, adipogenic differentiation and chemotactic activity. (**a**) Bar graph shows that the clonogenic capacity of ASC cultures is not influenced by the stimulation with 10% PL and the selective blocking for 48 hours of NPY receptors antagonists. %CFE (Colony forming efficiency) has been expressed as N. secondary colonies/N. plated cells x 100. N = 5. (**b**) Similarly, the rate of adipogenic differentiation of ASCs remains unaltered. The rate (%) of adipogenic differentiation has been calculated as: (N. adipogenic cells/N. Total cells) x 100. N = 5. (**c**) The chemotactic ability of ASCs is significantly enhanced by selective blocking of Y1, Y2 and Y5 receptors after stimulation with 10% PL. *p < 0.05, **p < 0.01, ^#^p < 0.0001. N = 4. Representative images of migrated ASCs stained with Giemsa after stimulation with DMEM, 10% PL alone or in presence of NPY receptor antagonists. Magnification 10×. PL, platelet lysate. NPYRAnts, NPY receptor antagonists.

Next, in order to prevent the response of ASCs to the PL-derived NPY, we have specifically inhibited the exogenous NPY action by blocking the three main receptors on cells surface through a combined pre-treatment with selective Y1, Y2 and Y5 competitive antagonists^4,31,32^. The targeted inhibition of the three receptors did not significantly influence the clonogenic efficiency and the differentiation capacity of ASCs towards the adipogenic lineage after treatment with 10% PL (Fig. 3a,b), suggesting that NPY is not the determining factor for these processes.

Interestingly, selectively blocking NPY receptors significantly enhanced the chemotaxis capacity of ASCs both compared to the stimulation with 10% PL and to non-stimulated controls (DMEM. Figure 3c, p < 0.05 and p < 0.001, respectively). The treatment with 10% PL was able to significantly increase chemotactic efficiency of ASCs compared to DMEM as expected (Fig. 3c, p < 0.01). The acquisition of the enhanced migratory activity of ASCs upon the combination of 10% PL and receptors antagonists for NPY was not ascribable to an altered cell proliferation (Supplementary Fig. 3d).

**Figure 4:**
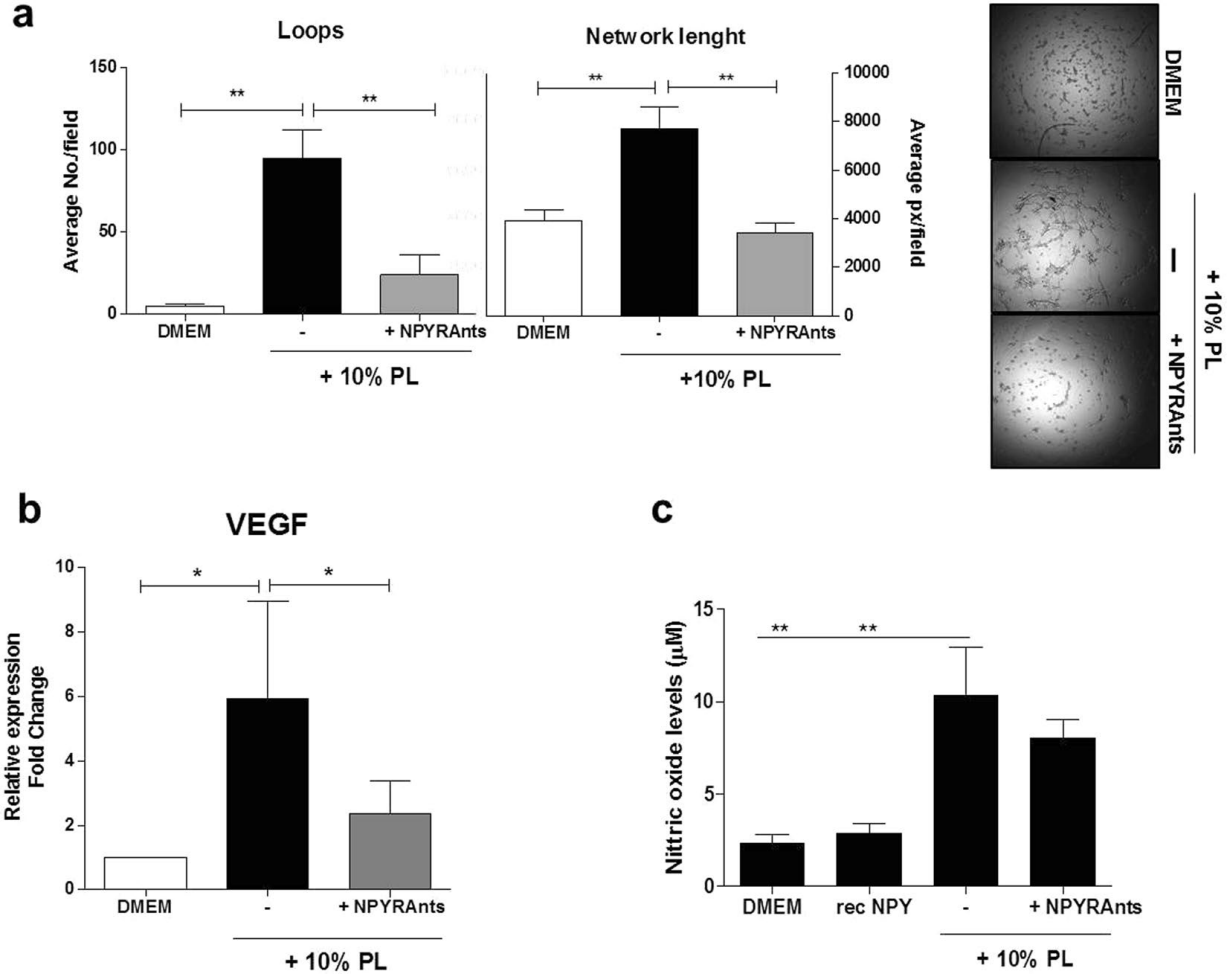
(**a**–**c**) Angiogenesis assay, VEGF expression and detection of nitric oxide soluble NO levels (**a**) Bar graph shows the decrease *in vitro* number of loops and network length found in Matrigel assays of ASCs cultured in 10% PL combined with or without NPY receptor antagonists, **p < 0.01. N = 5–6. Representative optical images of *in vitro* angiogenesis of ASCs. Magnification 4×. (**b**) Bar graph shows mRNA levels of VEGF detected by qPCR after stimulation with 10% PL and parallel blocking of NPY receptor in ASC cultures, compared to unstimulated control. N = 3. *p < 0.05. (**c**) Bar graph shows no significant alterations in the release of NO after blocking of NPY receptors with selective antagonists. However, 10% PL is able to significantly enhance the levels of NO respect to both unstimulated controls (DMEM) and recombinant NPY (10^−9^ M). N = 6. **p < 0.01. (rec NPY, recombinant NPY; PL, platelet lysate; NPYRAnts, NPY receptor antagonists.

More importantly, PL-induced angiogenesis *in vitro* (Fig. 4a, p < 0.01 vs DMEM, the unstimulated control) was reduced by blocking NPY receptors, as demonstrated by decreased number of both loops and network length generated by ASCs seeded onto Matrigel (Fig. 4a, p < 0.01). Notably, the degree of the reduced angiogenesis in presence of NPY receptors antagonists was comparable to unstimulated controls (Fig. 4a, p > 0.05). When cultures were supplemented with increasing concentrations of PL (15 and 20%) in presence of NPY receptor antagonists, the angiogenesis reduction effect was preserved only up to 15% PL (Supplementary Fig. 4d), indicating a partial concentration dependent effect. The stimulation with 10% PL was able per se to significantly increase the expression of VEGF mRNA compared to non-stimulated controls (Fig. 4b, p = 0.029), and more importantly the selective blocking of NPY receptors reduced VEGF mRNA levels in ASCs respect to 10% PL (Fig. 4b, p = 0.015), showing comparable levels to unstimulated controls. VEGF is a major angiogenic growth factor and is activated downstream of NPY signalling pathways^1,8,20,22^. The reduction in angiogenesis did not appear to be due to changes of NO levels released in ASC-derived conditioned media (Fig. 4c), whose production is reported to be upregulated by NPY^1^. However, in our experimental conditions the supplementation with the sole recombinant NPY did not alter NO levels, which were similar to unstimulated controls, although both significantly lower than those released after stimulation with 10% PL (Fig. 4c, both p < 0.001).

**Figure 5:**
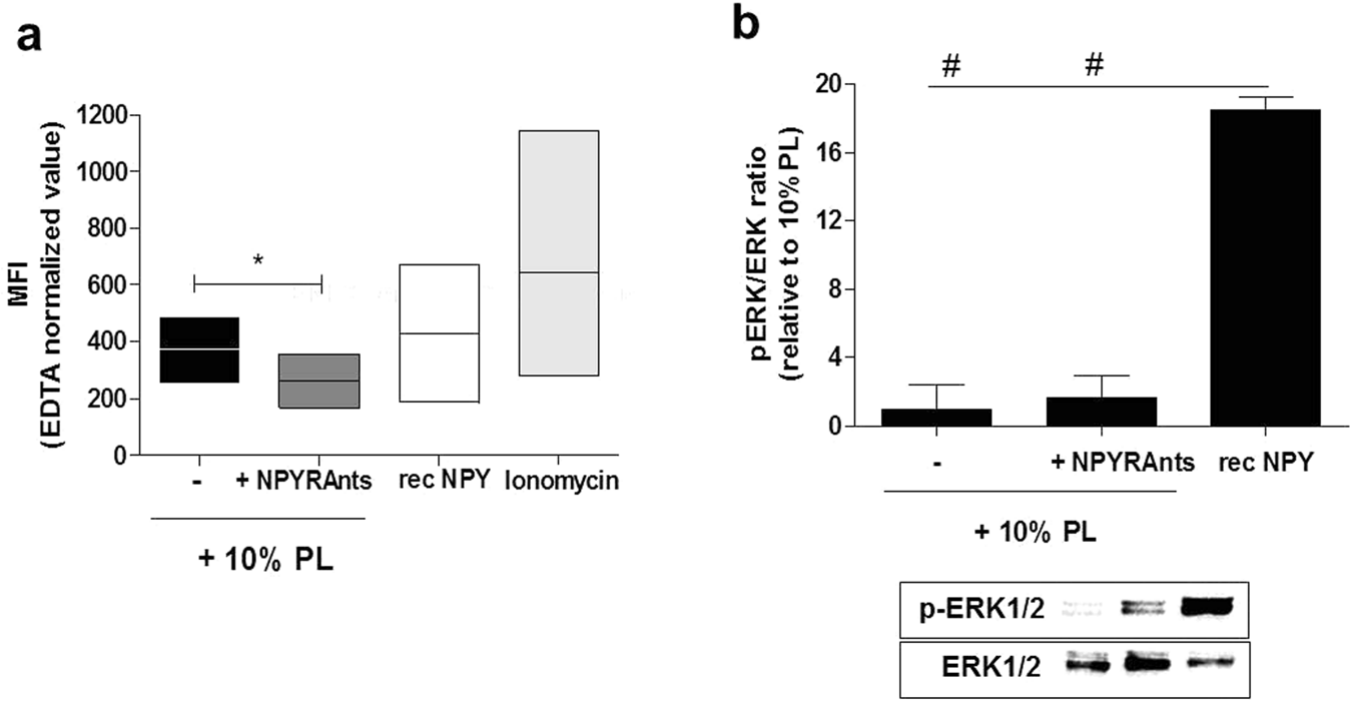
(**a**,**b**) Flow cytometry Analysis of ASC cultures stained with Fluo4AM and Western Blot Analysis of ERK signalling. (**a**) Boxplot displays the mean green fluorescence intensity of ASCs stimulated for 48 hours with 10% PL in presence of the selective antagonists for NPY receptors is significantly decreased compared to the treatment with 10% PL. The stimulation with recombinant NPY and Ionomycin have been used as biological and positive control, respectively. N = 3 biological replicates. *p < 0.05 (two-way ANOVA and Fisher LSD post-test). MFI, mean fluorescent intensity; PL, Platelet lysate; NPYRAnts, NPY receptor antagonists; rec NPY, recombinant NPY. (**b**) Bar graph shows that no difference in ERK phosphorylation is found in ASCs cultures after 48 hour of treatment with 10% PL combined to NPY receptor antagonists compared to the 10% PL. Nevertheless, the stimulation with recombinant NPY (10^−9^ M) is able to induce a significant increase of phospho ERK with respect to both treatments. N = 6. ^#^p < 0.001. Densitometry is presented as the ratio (pERK 1/2/Total ERK) normalized to control (10% PL). Cropped images are from samples ran on the same gel. Full-length blots are shown in Supplementary Fig. 5c. PL, platelet lysate; NPYRAnts, NPY receptor antagonists.

As both chemotaxis and angiogenesis are regulated by calcium signal and NPY has been reported to increase intracellular calcium levels^33,34^, we have investigated whether the PL-dependent NPY inhibitory and regulatory effect on angiogenesis and chemotaxis, might involve alterations of intracellular calcium levels. We have found a significant decrease in the calcium sensitive probe Fluo-4AM staining of ASCs when NPY is antagonized by selective blocking of its receptors compared to PL alone (Fig. 5a, p = 0.028), indicating the ability of PL-derived NPY to regulate intracellular calcium stores upon stimulation with PL.

Moreover, given that ERK phosphorylation is reported to be induced by NPY^35–38^ especially during chemotaxis^39,40^ and that the intracellular signalling cascade of NPY^12^ converging on ERK is also able to amplify growth-promoting effects^34^, we asked if ERK phosphorylation was altered after blocking of NPY. Our data have shown that the blocking of NPY receptors did not alter ERK phosphorylation in ASCs respect to 10% PL (Fig. 5b, p > 0.05), suggesting a negligible contribution of NPY to the ERK signalling pathway. However, the stimulation with recombinant NPY did significantly increase ERK phosphorylation either respect to 10% PL and when in combination with NPY receptor antagonists (Fig. 5b, both p < 0.0001).

**Figure 6:**
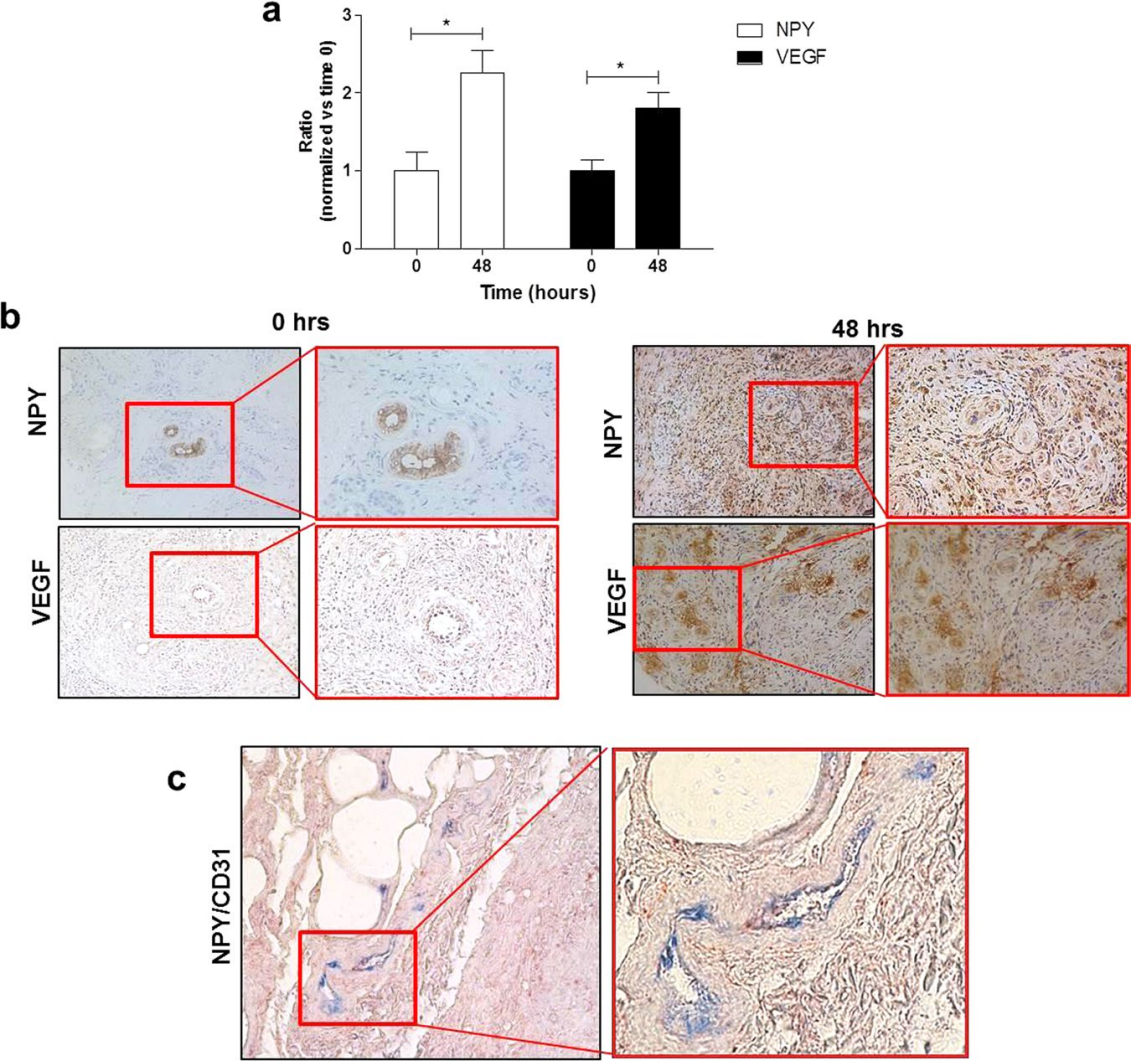
(**a**–**c**) Representative light microscopic appearance and staining for NPY, VEGF and CD31 of difficult wound biopsies from patients treated with leuco-platelet concentrates. (**a**) Bar graph showing the quantification of the expression of NPY and VEGF, which is significantly and concurrently enhanced after 48 hours of treatment. *p < 0.05. Results are expressed as the following ratio = No. Positive foci of angiogenesis/No. total foci of angiogenesis and normalized *vs* time 0. N = 3. (**b**) Staining for VEGF and NPY in patients difficult wound microscopic preparations. NPY is mainly distributed at the sites of angiogenesis including areas where it colocalizes with CD31 (**c**) a marker of mature endothelial cells. A similar localization of VEGF was also observed. Cell nuclei are counterstained with haematoxylin/eosin. In (**c**) CD31 stains blue and NPY red. Magnification 10X and 40X in b and c, respectively. The insert shows a higher magnification (20X and 60X in b and c, respectively).

Finally, in order to confirm that the employment of PL-based preparations *in vivo* could foster an increase of NPY, we have focused on chronic wounds of patients that exhibit impaired healing and where PL is successfully employed as regenerative tool to enhance skin repair and local angiogenesis^14,23,41,42^. Specifically, we have selected human biopsy samples obtained from the edge of lesion of difficult wounds (defined as hard-to-heal or chronic wounds), which previously have been clinically treated with the autologous leuco-platelet concentrate, a PL-like preparation. Immunohistochemistry analysis on these biopsies has shown that after 48 hours of treatment with PL-like concentrates, the expression of NPY is increased in the ulcer and mainly localized at the sites of angiogenesis within newly formed capillaries, which are characterized by a cubic and reactive endothelium (Fig. 6a,b, p = 0.029). The colocalized expression of NPY and CD31, a marker of mature endothelial cells, was also observed (Fig. 6c). Similarly, the increase of VEGF after 48 hours of treatment occurs in parallel (Fig. 6a,b, p = 0.016), therefore indicating an enhanced local angiogenesis.

## Discussion

In this study for the first time we show that PL is a non-neural and endogenous source of NPY, which acts as active bioavailable vehicle on ASCs. Accordingly, in our experimental settings, NPY also originates from ASCs cultures, which respond to the stimulus (PL) either by increasing intracellular protein level and excreting NPY in the microenvironment. Thus, it is conceivable that a self-reinforcing loop of NPY upon stimulation with PL is produced by ASCs. Although, the ability to source NPY is not specific to stromal cells as already demonstrated^26^, however the stroma (the structural component of several stem cells niches and tissues) is a preferential target of NPY when released in the microenvironment^28^. Interestingly, the stimulation of ASCs with PL does not entail a specific upregulation of the three main receptors for NPY. This result indicates a potential synergism between receptors in response to the stimulus, leading us to consider that PL could influence more the activity or the function of Y1, Y2 and Y5 rather than the sole protein levels, as also reported in literature^43^. In fact, Y1, Y2 and Y5 expressed on ASCs are functional, as their simultaneous and selective blocking impacts both chemotaxis and angiogenesis but adipocyte differentiation and clonogenic ability. Platelet lysate is enriched with additional growth factors and mitogens, which equally preserve adipocytic differentiation of stromal cells, thus likely masking the effects of shutting down NPY by selective blocking of its receptors. Thus, the potential synergism between NPY and other soluble molecules contained in PL cannot be fully ruled out. Despite this, angiogenesis is significantly reduced by inhibiting NPY receptors in ASCs, even in presence of the additional growth factors in PL preparations, therefore strengthening the specificity of the paracrine and angiogenic action of PL-derived NPY on ASCs. In particular, our data indicate that the reduction of the extent of angiogenesis is restored to the unstimulated control level. This result suggests that NPY could represent the major player promoting PL-mediated angiogenesis. Accordingly, it has been demonstrated that the specific angiogenic contribution of NPY upon ischemia mainly originates from platelets, therefore clearly validating NPY as critical factor for revascularization upon damage conditions^11^. The angiogenic property of NPY has been well described in endothelial and vascular smooth muscle cells^8^ and in several tissues^3,44^. In this study, we implement the knowledge regarding the biological action of NPY on mesenchymal stem cells and specifically on the adipose progenitors instead of the mature adipose tissue or BMMSCs^17,18^. Notably, the origin of ASCs has been hypothesized from the stromal vascular fraction and specifically from the pericytes which are angiogenic populations closely associated to adipose progenitors^20,22,45^. In addition, the development of the adipose stromal population strictly depends on angiogenesis and ASCs are able to secrete growth factors^22^. The PL-derived NPY and that originated from ASCs in response might contribute to preserve this function in adipose progenitors. Interestingly, it has been reported that the pre-treatment of the mesenchymal cellular fraction with NPY prior to *in vivo* transplantation in murine models of myocardial infarction, increases cardiac differentiation of stromal cells combined with enhanced angiogenesis and amelioration of cardiac function^16^. However, to the best of our knowledge, no studies are currently investigating the angiogenic effects mediated by PL-derived NPY on ASCs. Notably, Tilan *et al*. have recently shown in a murine model of ischemia that the contribution of NPY during the late stages of recovery are dependent on the release of NPY from megakaryocyte/platelets activation, resulting in increased soluble levels of NPY in the systemic circulation and vascularization at the sites of angiogenesis^11^. Although it has been only demonstrated in endothelial and vascular smooth muscle cells, in line with our data this study strengthens the notion that NPY originating from platelets is able to increase cells angiogenic potential. In addition, adipose tissue-derived angiogenesis has been directly linked to the activation of NPY subtype receptors^46^, confirming that the angiogenic action of NPY is not limited to the neural, endothelial and vascular system^47,48^. Moreover, in our study we have highlighted the strict dependence of VEGF from NPY. Neuropeptide Y is upstream of VEGF signalling^47^, as well as either PL-based preparations and platelet activation have been implicated into the release of stored factors driving angiogenesis, including VEGF^19–22,49,50^. Upon ischemic damage, the activation of NPY subtype receptors triggers angiogenesis in endothelial cells through NO and release of VEGF^8,48^. Thus, the parallel decrease of angiogenesis and VEGF in ASCs after preventing the cross talk between cells and PL-derived NPY, recapitulates these effects. However, difference in NO soluble levels was not observed, likely due to the lack of a vascular insult, but also by the fact that in *in vivo* models of angiogenesis ascribable to NPY, the increase of NO does not always parallel VEGF^8^. Additionally, our results also highlight that inhibition of NPY receptors increases chemotaxis. The NPY is reported to possess bimodal features. Accordingly, its biological action has been described not only in a concentration-dependent fashion^51^ but also associated to the relative affinity with its receptors in the microenvironment^17,34^. Higher concentrations of NPY have been reported to neither increase cell proliferation, nor adipocytes differentiation and migration in wound scratch *in vitro* assays^52,53^. Thus, the observation that ASCs chemotaxis is increased by preventing the cross talk between NPY and ASCs is in line with a more regulatory role of NPY subjected to modulation in an activity-dependent manner. Interestingly, in our dataset, NPY shut down through receptors inhibition, decreases the intracellular calcium in ASCs, indicating that PL is able to primarily perturb calcium stores in ASCs through NPY. We cannot exclude that additional intracellular proteins are able to similarly mediate this function in the stromal fraction. Nevertheless, we found that the blocking of NPY function in ASCs alters both angiogenesis and chemotaxis, two main processes strictly restrained by calcium levels^33^. The NPY is reported to raise intracellular calcium levels in vascular smooth muscle and endothelial cells^1,5,34,54^ and to modulate cardiac contractility^55,56^. Notably, in some cell systems such as the retinal ganglion cells, NPY attenuates the calcium increase^30^. Although our results show a non-involvement of the ERK pathway, the stimulation with recombinant NPY instead of PL-derived NPY, strictly induces ERK phosphorylation. This suggests that the modality by which NPY is conveyed (in the form of single agent or as part of a plethora of soluble mediators contained in PL), may determine the activation of different biological and molecular signalling pathways. In this regard, the same effect has been observed for the release of NO in ASC-derived conditioned media.

So far, the majority of studies including those on BMMSCs^17,18^ have investigated the biological role of NPY by exogenous treatment with the recombinant protein. In this study, we have taken advantage of the NPY endogenously contained in PL, therefore reproducing a more physiological scenario. Accordingly, our data highlight that similarly to PL, leuco-platelet preparations employed *in vivo* may contain NPY. The tissue is able to respond by increasing the foci of angiogenesis where the positivity to NPY and VEGF is maximized. Coherent with our *in vitro* data, this result strengthens the significant the tropic contribution of platelets-derived NPY in periphery to induce angiogenesis and certainly migration (two main repairing processes amplified and enhanced *in vivo* by the employment of PL on difficult wounds). The role of NPY in wound healing has been demonstrated in mouse models and associated to its pro- and anti-inflammatory features, fostering chemotaxis and angiogenesis^57^ and involving macrophage-derived NPY within the adipose tissue^58^, a powerful modulator of inflammation^22^. Nevertheless, the precise molecular mechanisms and the modality by which PL-derived NPY could act *in vivo* on adipose tissue and cross talk with the immune system through the mesenchymal fraction, requires further investigation.

Our study has some limitations. First, we have hampered the *in vitro* response of ASCs to NPY rather than blocking NPY in PL by employing a neutralising antibody. This latter approach could have helped us to deeper understand if NPY is a discriminating factor in mediating the effects of PL. Similarly, we couldn’t inhibit the PL-derived NPY in patients for ethical reasons, therefore we are not able to fully confirm that the *in vivo* enhancement of PL-mediated angiogenesis is ascribable to the sole NPY. Finally, we have highlighted the *in vitro* effects of PL-derived NPY on the stromal lineage. Consequently it would be important to verify the involvement of the stromal fraction also *in vivo*.

In conclusion, this study has identified a non-neural source of NPY, present in the haemoderivate PL, and confirmed the trophic and peripheral angiogenic action of the peptide^59^. This feature could be exploited in wound healing as well as in other pathological cardiovascular disorders, where the enhancement of angiogenesis is desirable even indirectly by acting on the stromal progenitors. The combination of NPY and other growth repairing factors contained in PL, could also lead to new developed formulations of PL-based preparations, where the level of NPY could be modulated according to clinical needs, tissue-specificity and cell type.

## Electronic supplementary material

Supplementary materials
