# Supplementary material for: Platelet Lysate-Derived Neuropeptide y Influences Migration and Angiogenesis of Human Adipose Tissue-Derived Stromal Cells
Source: Sci Rep. 2018 Sep 25;8:14365. doi: 10.1038/s41598-018-32623-8 (PMC6156505; doi:10.1038/s41598-018-32623-8)
Supplement: Supplementary file 1 — Supplementary materials [file 41598_2018_32623_MOESM1_ESM.pdf]

PLATELET LYSATE-DERIVED NEUROPEPTIDE Y INFLUENCES MIGRATION AND ANGIOGENESIS OF HUMAN ADIPOSE TISSUE-DERIVED STROMAL CELLS

Rita Businaro[1]*, Eleonora Scaccia[1]*, Antonella Bordin[1], Francesca Pagano[1], Mariangela Corsi[1], Camilla Siciliano[1], Raffaele Capoano[2], Eugenio Procaccini[3], Bruno Salvati[2], Vincenzo Petrozza[1], Pierangela Totta[4], Maria Teresa Vietri[5], Giacomo Frati[1,6], Elena De Falco[1]
*equal contribution

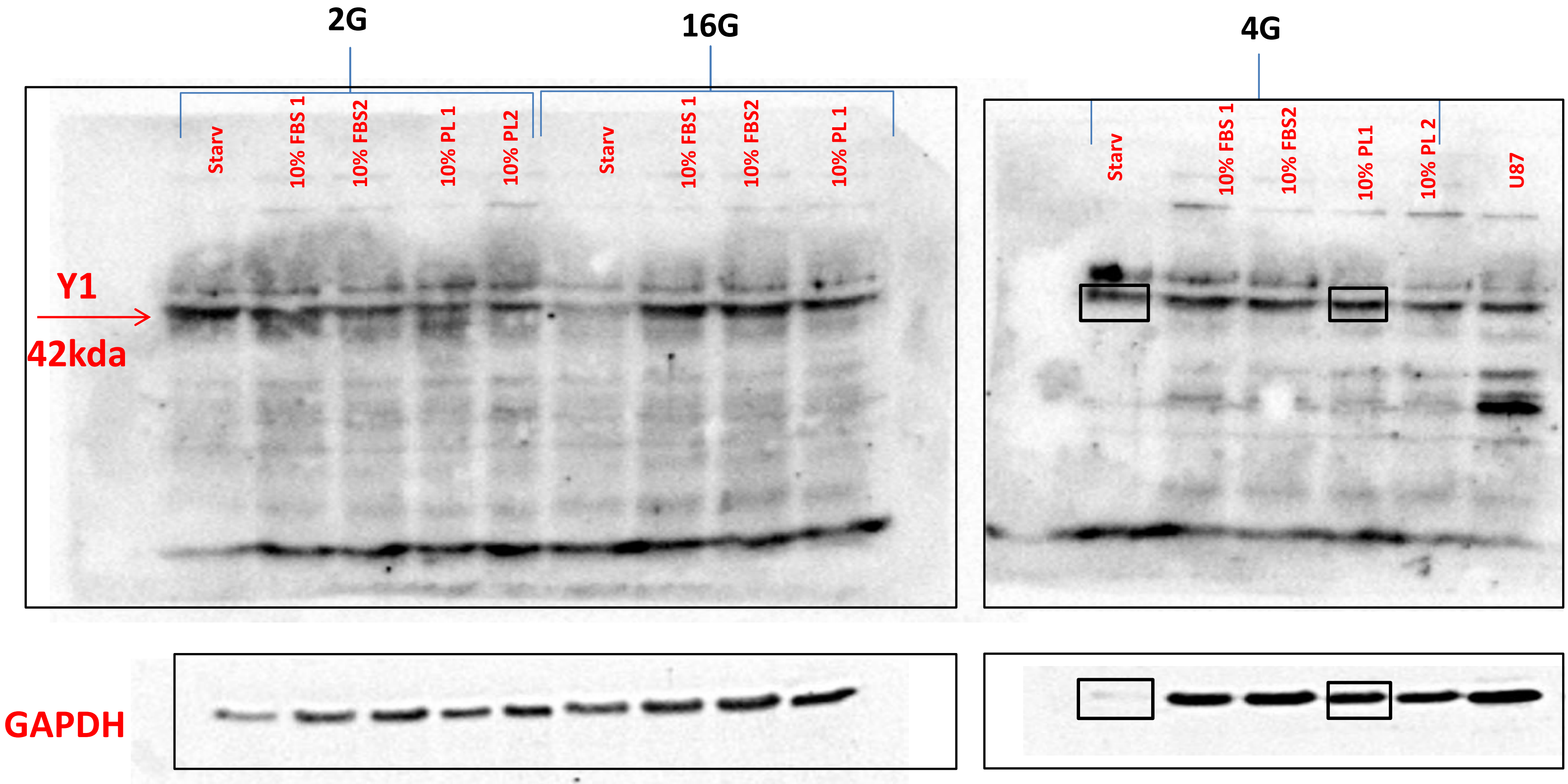

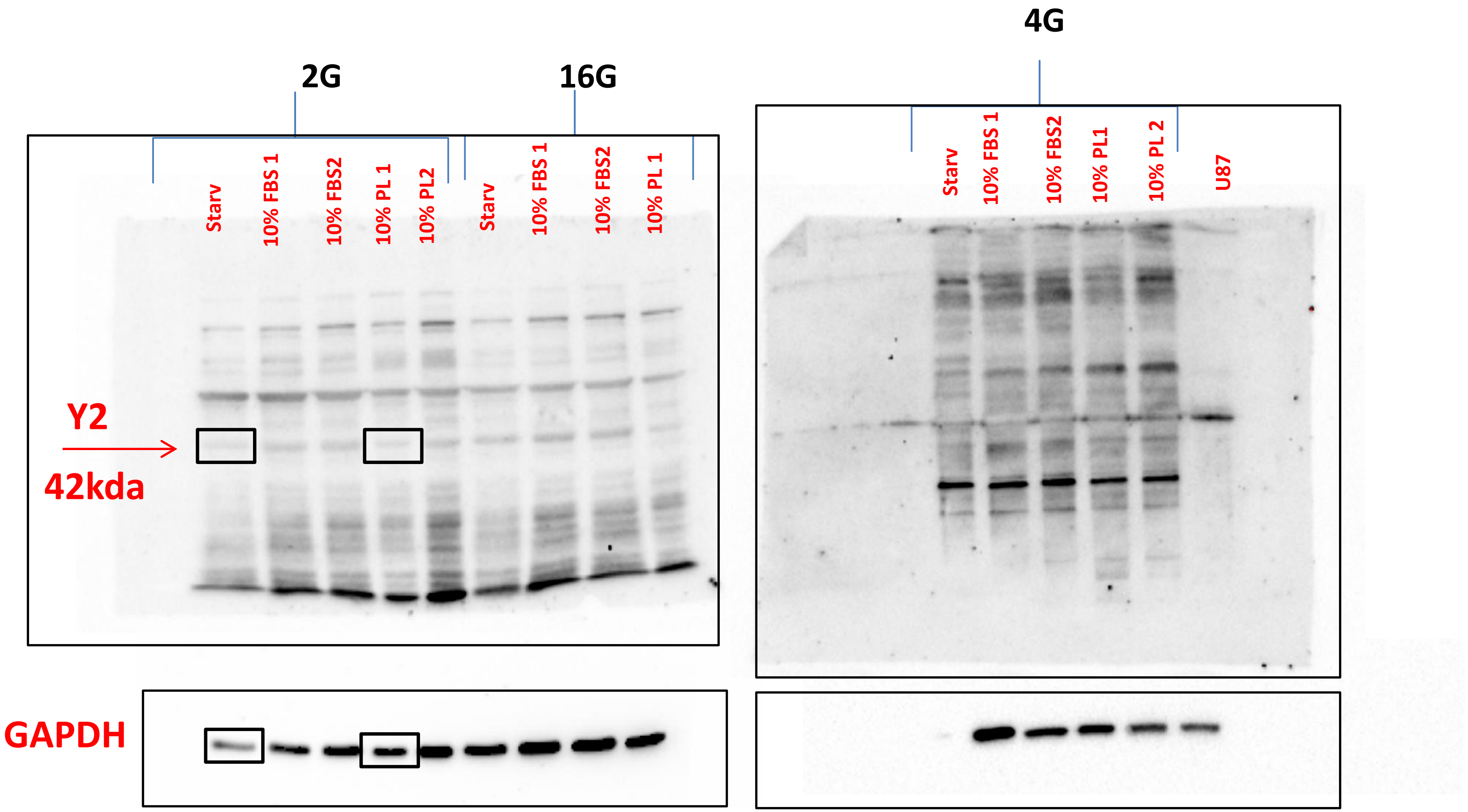
2G
16G
4G
Starv
10% FBS 1
10% FBS2
10% PL 1
10% PL2
Starv
10% FBS 1
10% FBS2
10% PL 1
Starv
10% FBS 1
10% FBS2
10% PL1
10% PL 2
U87
Y2
42kda
GAPDH

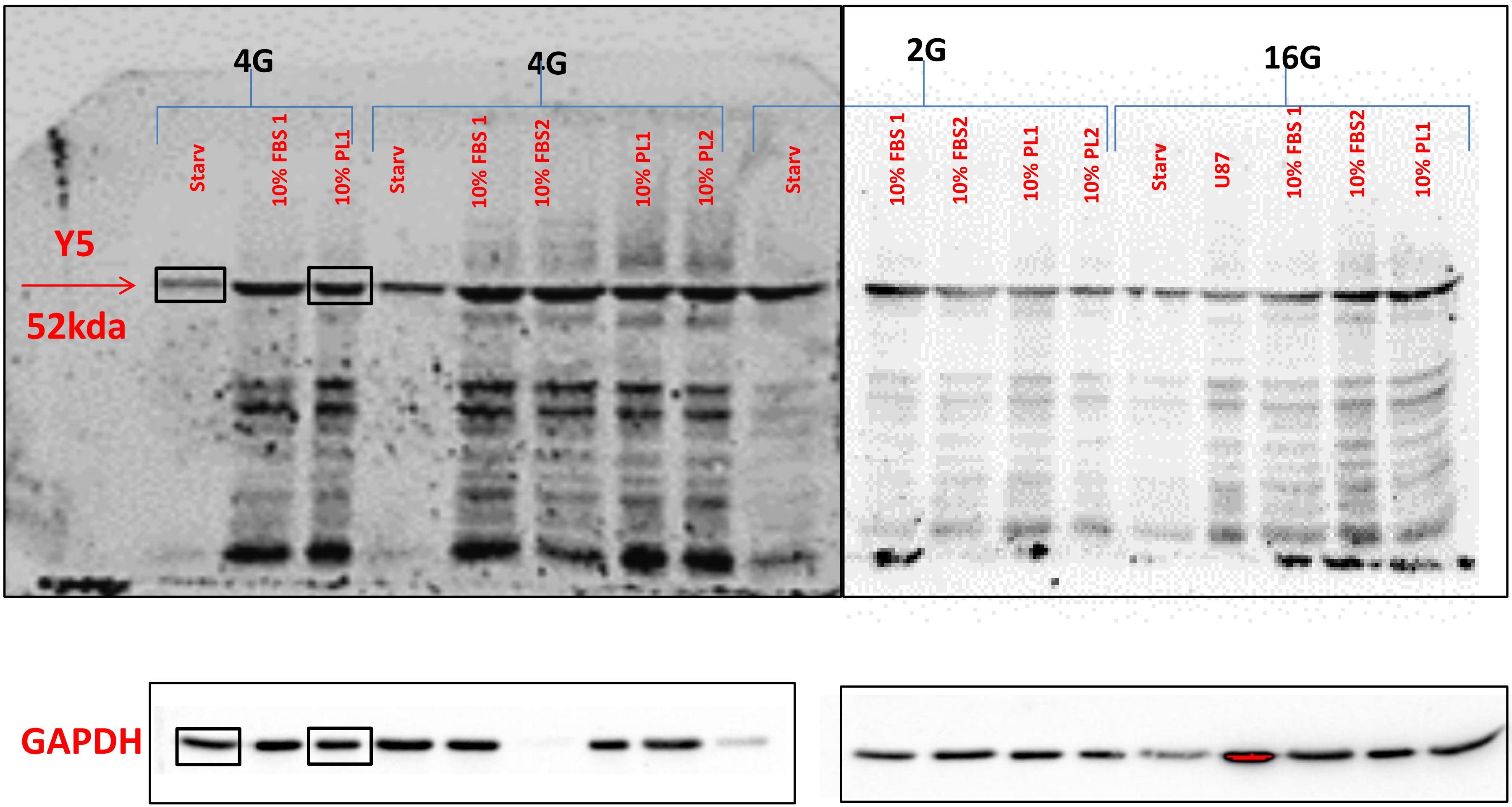

Supplementary Figure 1d. Uncropped western blots for Y1, Y2 and Y5 receptors of adipose stromal cell-derived total cell extracts. GAPDH was the loading control, whereas U87 cell line and ASCs treated with FBS were used as positive and biological controls. All samples were ran on two gels. Y1 and Y5 were blotted on the same membrane. PL, platelet lysate, FBS, foetal bovine serum, starv, starvation.

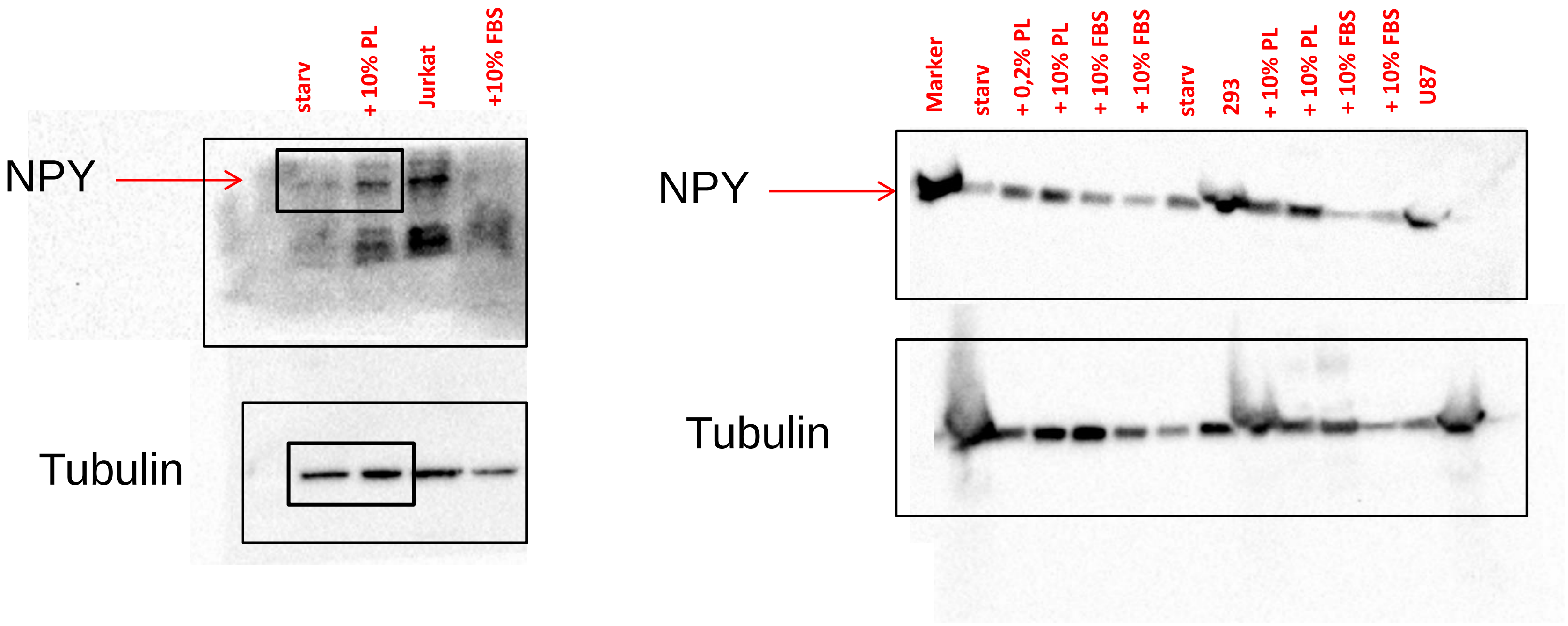

Supplementary Figure 2c. Uncropped western blots for NPY of ASC-derived total cell extracts. Tubulin was the loading control. Jurkat, 293 and U87 and ASCs treated with FBS were used as references. PL, platelet lysate.

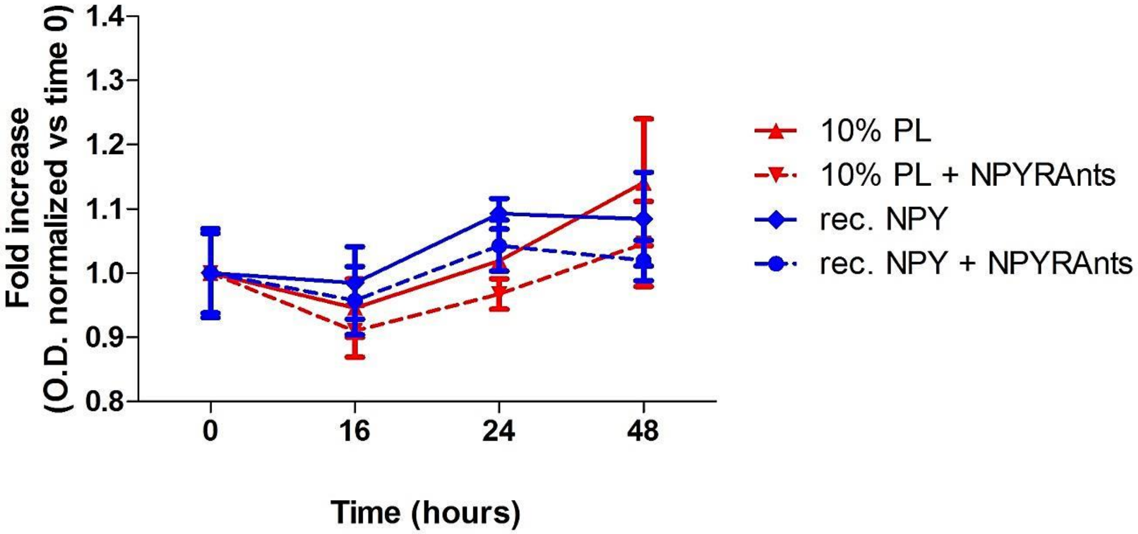

Supplementary Figure 3d. Cell Proliferation assay. This test performed by MTS shows no alteration in the mitogenesis of ASC cultures after 48 hours of stimulation with all treatments. PL, platelet lysate; rec. NPY, recombinant NPY; NPYRAnts, NPY receptor antagonists. O.D., optical density

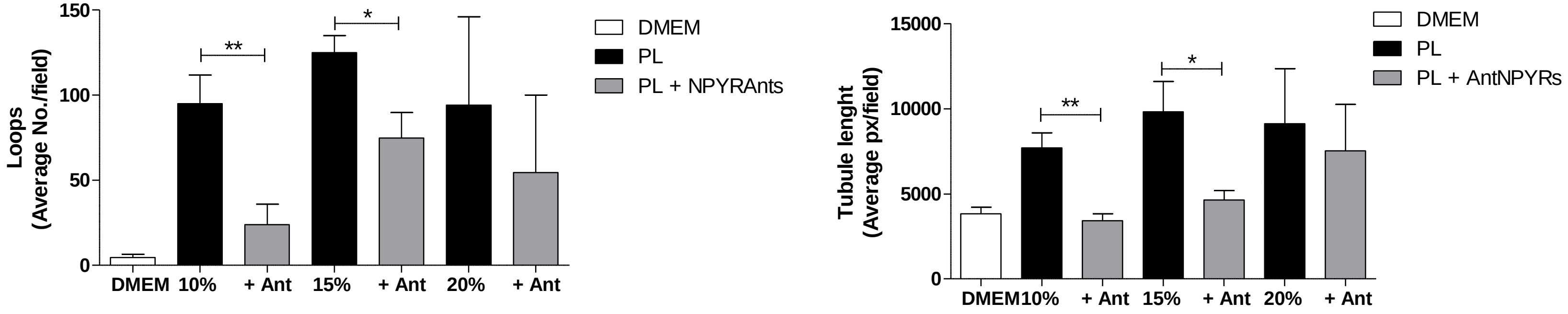

Supplementary Figure 4d. Matrigel assays on ASCs stimulating with increasing concentrations of platelet lysate (10, 15 and 20%) alone or in combination with NPY receptor antagonists. Results show that a significant reduction of the in vitro number of loops and network length in presence of NPY receptor antagonists is preserved up to 15% PL. PL, platelet lysate. NPYRAnts, NPY receptor antagonists. *p<0.05, **p<0.01.

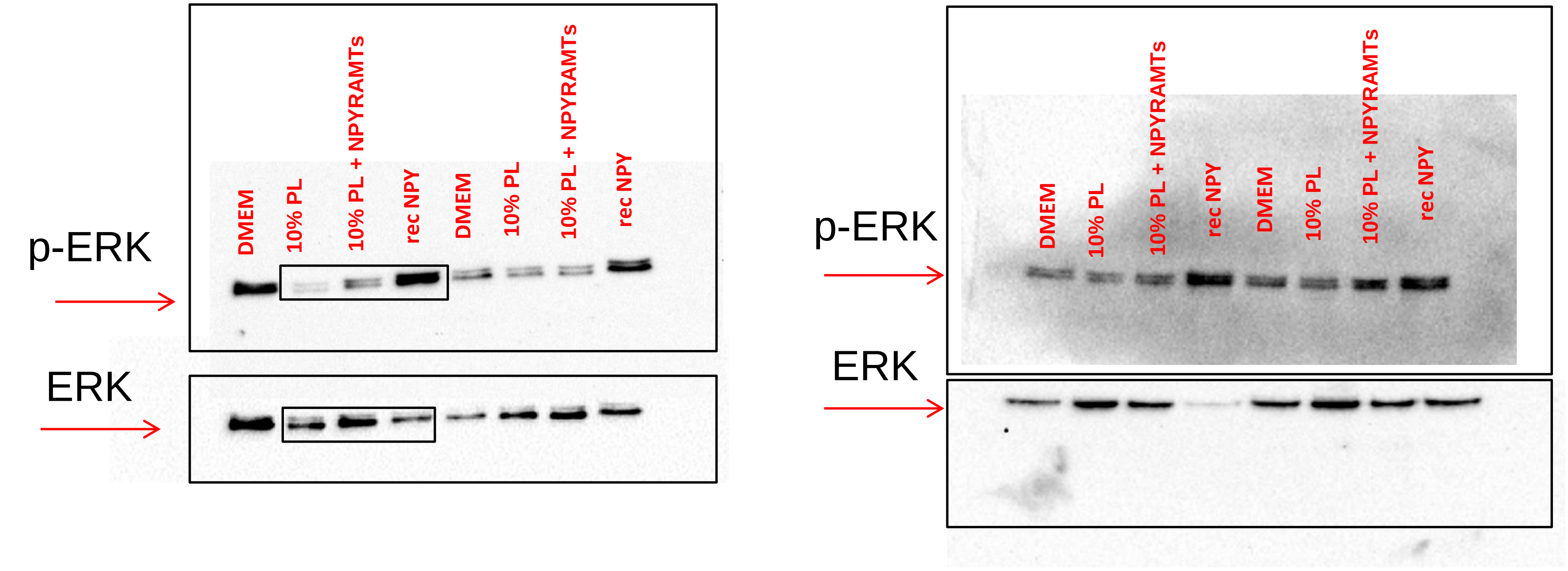

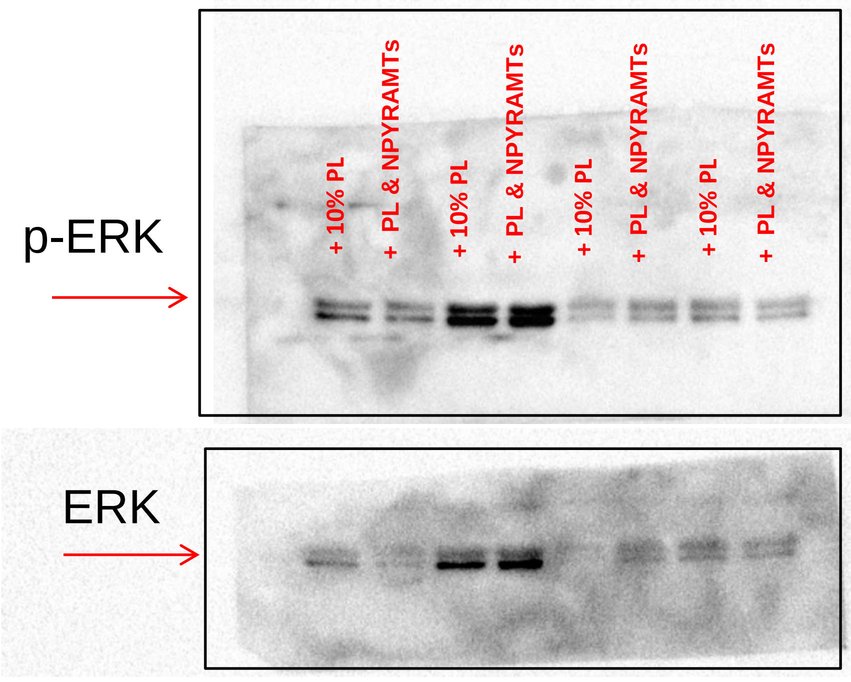

Supplementary Figure 5c. Full-length Western Blots of phospho-ERK1/2 of all ASC cultures stimulated after 48 hours with Platelet lysate, in combination with selective NPY receptors antagonists or recombinant NPY. The graph shows that no difference in ERK phosphorylation is found between treatments. However, the stimulation with recombinant NPY (10^{-9}M) significantly enhances the protein levels of phospho ERK. PL, platelet lysate; NPYRAnts, NPY receptor antagonists, rec NPY, recombinant NPY.
